# Diabetes mellitus duration and mortality in patients hospitalized with acute myocardial infarction

**DOI:** 10.1186/s12933-022-01655-w

**Published:** 2022-10-29

**Authors:** Marta Baviera, Stefano Genovese, Pierluca Colacioppo, Nicola Cosentino, Andreana Foresta, Mauro Tettamanti, Ida Fortino, Maria Carla Roncaglioni, Giancarlo Marenzi

**Affiliations:** 1 Lab of Cardiovascular Prevention Dipt of Health Policy, Istituto di Ricerche Farmacologiche Mario Negri IRCCS, Via Mario Negri 2, 20156 Milan, Italy; 2 Centro Cardiologico Monzino IRCCS, Milan, Italy; 3 Laboratory of Geriatric Neuropsychiatry, Istituto di Ricerche Farmacologiche Mario Negri IRCCS, Milan, Italy; 4 Lombardy Region, Regional Health Ministry, Milan, Italy

**Keywords:** Acute myocardial infarction, Diabetes mellitus, Diabetes duration, In-hospital mortality, 1-year mortality

## Abstract

**Background:**

Diabetes mellitus (DM) is associated with an increased mortality risk in patients hospitalized with acute myocardial infarction (AMI); however, no studies have investigated the impact of the duration of DM on in-hospital mortality. In this study, we evaluated in-hospital mortality in AMI patients according to DM status and its duration.

**Methods:**

Using health administrative databases of Lombardy, DM patients≥50 years hospitalized with AMI from 2010 to 2019 were included in the analysis and were stratified according to the duration of DM: <5, 5–10, and > 10 years. The primary endpoint was mortality during AMI hospitalization and the secondary endpoint was 1-year mortality in comparison with No-DM patients. Logistic and Cox regressions analyses were used to estimate odds ratios (ORs, CI 95%) and hazard ratios (HRs, CI 95%) for the outcomes, according to DM status and duration and AMI type (STEMI and NSTEMI).

**Results:**

Our study cohort comprised 29,566 and 109,247 DM and No-DM patients, respectively. Adjusted ORs and HRs showed a significantly higher risk of in-hospital mortality (OR 1.50, 95% CI 1.43–1.58) and 1-year mortality (HR 1.51, 95% CI 1.46–1.55) in DM patients in comparison with those without. These risks increased progressively with the duration of DM, with the highest risk observed in patients with DM duration ≥ 10 years (OR 1.59, 95% CI 1.50–1.69 for in-hospital mortality and HR 1.59, 95% CI 1.53–1.64 for 1-year mortality). These findings were similar in STEMI and in NSTEMI patients.

**Conclusions:**

Our study demonstrates that the duration of DM parallels mortality risk in patients hospitalized with AMI, highlighting that DM duration should be considered as an important early prognostic risk factor in patients with AMI.

**Supplementary Information:**

The online version contains supplementary material available at 10.1186/s12933-022-01655-w.

## Introduction

Diabetes mellitus (DM) is a frequent comorbidity among patients hospitalized with acute myocardial infarction (AMI) [1–3]. In this clinical setting, the presence of DM has been systematically associated with a higher in-hospital mortality [1–7]. Despite evidence for major improvements in outcomes over the past 40 years in the general AMI population, regardless of DM status, a two-fold higher in-hospital mortality rate in DM patients has been consistently reported across decades [4, 5].

Previous studies addressing the prognostic relevance of DM in patients hospitalized with AMI have considered DM as a dichotomous variable (yes vs. no) and only a few assessed the risk of in-hospital death in relation to known or unknown DM, chronic glycemic status, as estimated by the glycated hemoglobin, and anti-hyperglycemic therapy (oral vs. insulin) before index hospitalization [1–12]. However, to the best of our knowledge, no studies investigated the association between DM duration and in-hospital mortality in AMI patients. Notably, the duration of DM has been shown to closely reflect microvascular and macrovascular complication burden, which, in turn, is associated with in-hospital clinical outcome in AMI patients [3, 13–15]. Current guidelines have recently considered a long duration of DM (≥10 years) as a critical modifier when assessing cardiovascular risk in DM patients [15]. However, whether DM duration, by summarizing the patient’s burden of DM-related comorbidities, reflects in-hospital mortality of patients with AMI has never been investigated yet. Therefore, in this study, we used administrative data from the most populated Italian region - Lombardy - to evaluate in-hospital mortality in a large unselected population of AMI patients according to DM status and its duration. Furthermore, given the different in-hospital mortality risk between patients with ST-elevation myocardial infarction (STEMI) and non-ST-elevation myocardial infarction (NSTEMI) [16], we also analyzed the prognostic impact of DM duration in these two types of AMI, considered separately.

## Methods

### Data source

Our study used linkable administrative health databases of the Lombardy region in (Italy), which includes a population registry with demographic data of all residents and detailed information on hospital records and drug prescriptions reimbursed by National Health System. Data are available for about 10 million inhabitants of Lombardy from 2000 to 2019. Access to data is allowed within the agreement between the Istituto di Ricerche Farmacologiche Mario Negri (IRFMN) and Regional Health Ministry of Lombardy. Healthcare in Italy is publicly funded for all residents, irrespective of social class or employment, and everyone is assigned a personal identification number kept in the National Civil Registration System. All residents are assisted by general practitioners and are covered by the National Health System (NHS) with high level of completeness regarding drug prescriptions, diagnosis, and length of observation. The pharmacy prescription database contains the medication name and anatomic therapeutic chemical classification code (ATC), quantity, and date of dispensation of drugs reimbursed by the NHS. The hospital database contains information on date of admission, discharge, death, primary diagnosis, and up to five co-existing clinical conditions and procedures performed. The diagnoses, uniformly coded according to the 9th International Code of Diseases (ICD-9-CM) and standardized in all Italian hospitals, are compiled by the hospital specialists directly in charge of the patients and are validated by hospitals against detailed clinical-instrumental data, as they determine reimbursement from the NHS.

A unique identification code allows linkage of all databases. To ensure privacy, each identification code was automatically converted into an anonymous code before we received the dataset. In Italy studies using retrospective anonymous data from administrative databases that do not involve direct access by investigators to identification data do not require Ethics Committee/IRB approval or notification nor patient informed consent signing.

### Study cohorts

Patients 50 years and older with a hospitalization due to AMI (both ST-elevation [STEMI] and non-ST-elevation [NSTEMI] myocardial infarction [ICD-9-CM codes 410.x]) from January 1, 2010, through December 31, 2019, were included in the analyses. Patients were divided in two groups according to DM status at time of hospitalization for AMI. DM was defined as chronic exposure to anti-hyperglycemic agents (at least two prescriptions of ATC code A10* within the same calendar year). Patients with DM were stratified into three groups according to disease duration, estimated using first exposure to anti-hyperglycemic agents: <5 years, 5–10 years, and > 10 years. Index date for cohort entering was the date of AMI.

### Study variables

The most prevalent complications during AMI hospitalization and the history of comorbidities of interest in the ten years before the index date were retrieved using hospital records (up to six co-existing diagnosis and procedures). Exposure to anti-hyperglycemic drugs and other medications of interest in the 12 months before index date were also retrieved (Appendix).

#### Study outcomes and follow-up

The primary outcome of the study was in-hospital mortality. As DM patients with AMI continue to be at increased risk of death after hospital discharge, particularly in the first year [4–6], we also analyzed one-year all-cause mortality from index date as secondary outcome. Patients were followed-up from the index date until death, migration or up to the end of one-year follow-up.

### Statistical analysis

Baseline characteristics were evaluated using descriptive statistics. Categorical variables were described using frequencies and percentages and compared using Chi-square test; continuous variables were described using mean and standard deviation (SD) and compared using Student’s t-test. Baseline characteristics were also reported according to the duration of DM (< 5 years, 5–10 years, and > 10 years) in the overall AMI population and in patients with STEMI and NSTEMI considered separately. The effect of the DM duration was assessed using p for trend.

Crude incidence rates were calculated as the number of events divided by the total number of person-years at risk and expressed per 100 person-years with 95% confidence interval (CI). Differences in cumulative incidence of 1-year mortality were plotted using Kaplan–Meier curves, both for comparison between DM vs. No-DM patients and according to DM duration. Kaplan–Meier curves were also reported for STEMI and NSTEMI subgroups.

Logistic regressions were used to estimate odds ratios (ORs) and 95% CIs for primary outcome and for each comparison according to DM status and its duration. ORs were also calculated for STEMI and NSTEMI subgroups and for each comparison.

Cox proportional hazard regression model was used to estimate hazard ratios (HRs) and 95% CI for 1-year mortality, comparing DM vs. No-DM patients and according to DM duration. HRs were also reported for STEMI and NSTEMI subgroups. The proportionality assumptions for these models were checked using log-log plots.

ORs and HRs were adjusted according to an epidemiological model including the variables known to be most closely associated with in-hospital mortality in patients with AMI [17, 18] age, gender, prior AMI, AMI type (STEMI vs. NSTEMI), percutaneous coronary intervention (PCI) performed during index hospitalization, chronic heart failure, renal disease, peripheral vascular disease and cerebrovascular disease. Calibration of the logistic model was assessed by means of a calibration plot finding relatively small departures from predicted probability of in-hospital death.

Multicollinearity was assessed using Variance Inflation Factors (VIFs) for the variables included in the models finding rather low VIFs that ranged between 1.05 and 1.29.

We compared the Akaike Information Criterion (AIC) of the model with DM duration with that with DM presence to understand which models for in-hospital and one-year mortality were better.

Forest Plots were used to graphically represent ORs and HRs: these graphs show comparisons between DM versus No-DM patients for the whole cohort and for subgroups analysis.

All the analyses were performed using SAS version 9.4 (SAS Institute, Cary, NC, USA).

## Results

**Fig. 1:**
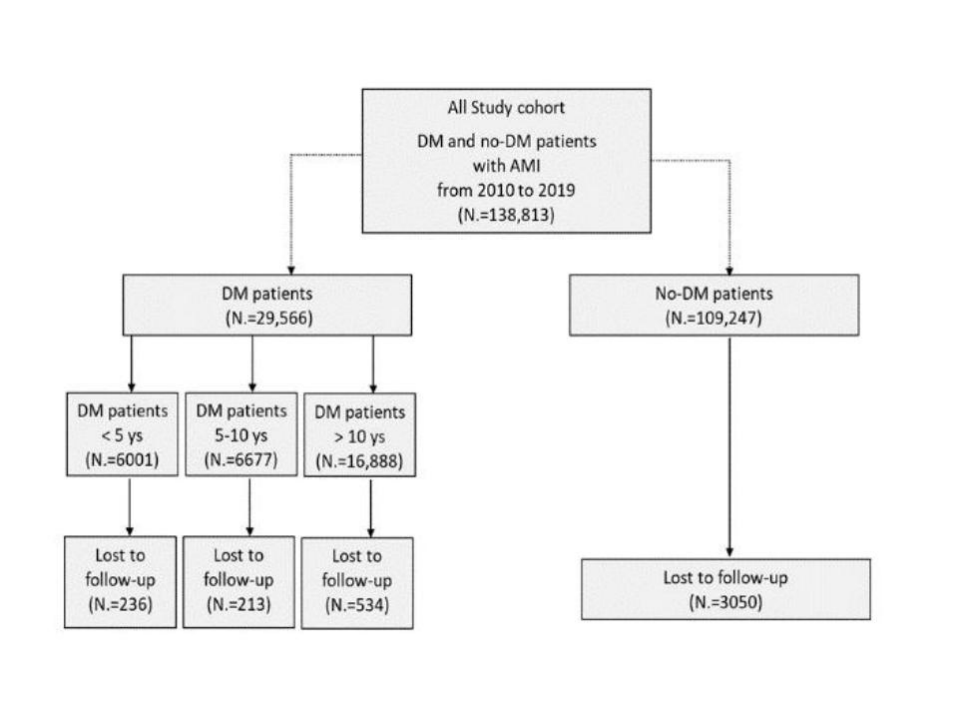
Flow diagram of patients enrolled in the study. AMI = acute myocardial infarction; DM = diabetes mellitus

A total of 138,813 patients hospitalized with AMI from 1 to 2010 to 31 December 2019 were included in the analysis; 29,566 patients had DM and 109,247 did not have DM. A flowchart of the study is provided in Fig. 1.

The baseline characteristics of the study population according to DM status and its duration are shown in Table 1. Patients with DM had more cardio-cerebrovascular comorbidities and were treated with a higher proportion of chronic cardiovascular medications in comparison with those without DM. Comorbidities and exposure to insulin and cardiovascular medications increased in parallel with DM duration. Table 2 shows in-hospital complications and procedures during index hospitalization for AMI. Patients with DM, especially those with the longest DM duration, developed more complications than patients without DM (all p < 0.0001). In comparison with no-DM patients, those with DM underwent less frequently PCI, with a decreasing rate with DM duration. Baseline clinical characteristics and in-hospital complications and procedures of patients hospitalized for either STEMI or NSTEMI, according to DM status and its duration, are reported in Supplementary material (Tables 1, 2, 3 and 4 S).

**Table 1:** Baseline characteristics of patients hospitalized with acute myocardial infarction according to diabetes mellitus status and its duration, from 2010 to 2019

	No-DM Patients No. (109,247)	DM Patients No. (29,566)	P Value	DM Patients (duration of DM) No. (29,566)					P for trend	
**Variables**				**< 5 years** No. (6001)	**5–10 years** No. (6677)	**> 10 years** No. (16,888)				
**Age** (years), mean ± SD	72.7 ± 11.9	71.7 ± 9.6	< 0.0001	71.1 ± 10.5	70.3 ± 10.0		72.5 ± 9.0		< 0.0001	
**Age groups** (years), n (%)										
50–64	31,376 (28.72)	7424 (25.11)	< 0.0001	1803 (30.04)	2098 (31.42)		3523 (20.86)		< 0.0001	
65–80	41,831 (38.29)	15,281 (51.68)		2723 (45.38)	3223 (48.27)		9335 (55.28)			
> 80	36,040 (32.99)	6861 (23.21)		1475 (24.58)	1356 (20.31)		4030 (23.86)			
**Gender** (female)	40,094 (36.70)	11,345 (38.37)	< 0.0001	1952 (32.53)	2311 (34.61)		7082 (41.94)		< 0.0001	
**AMI type**										
STEMI	52,753 (48.29)	9937 (33.61)	< 0.0001	2309 (38.48)	2494 (37.35)		5134 (30.40)		< 0.0001	
NSTEMI	56,494 (51.71)	19,629 (66.39)	< 0.0001	3692 (61.52)	4183 (62.65)		11,754(69.60)		< 0.0001	
**History of comorbidities**, n (%) (in the previous 10 years)										
Cerebrovascular disease	10,579 (9.68)	5501 (18.61)	< 0.0001	868 (14.46)	1028 (15.40)		3605 (21.35)		< 0.0001	
Prior myocardial infarction	4935 (4.52)	4289 (14.51)	< 0.0001	969 (16.15)	871 (13.04)		2449 (14.50)		< 0.0001	
Chronic ischemic heart disease	13,320 (12.19)	8307 (28.10)	< 0.0001	1370 (22.83)	1570 (23.65)		5358 (31.73)		< 0.0001	
Prior PCI or CABG	9839 (9.01)	6371 (21.55)	< 0.0001	1208 (20.13)	1317 (19.72)		3846 (22.77)		< 0.0001	
Chronic heart failure	1660 (1.52)	1206 (4.08)	< 0.0001	186 (3.10)	214 (3.21)		806 (4.77)		< 0.0001	
Atrial fibrillation	7394 (6.77)	3075 (10.40)	< 0.0001	546 (9.10)	684 (10.24)		1845 (10.92)		0.0003	
Peripheral vascular disease	4322 (3.96)	4746 (16.05)	< 0.0001	541 (9.02)	762 (11.41)		3443 (20.39)		< 0.0001	
Lower limb complication	975 (0.89)	1601 (5.42)	< 0.0001	163 (2.72)	197 (2.95)		1241 (7.35)		< 0.0001	
Renal disease	4581 (4.19)	3419 (11.56)	< 0.0001	417 (6.95)	519 (7.77)		2483 (14.70)		< 0.0001	
COPD	6209 (5.68)	2505 (8.47)	< 0.0001	502 (8.37)	543 (8.13)		1460 (8.65)		0.4201	
Cancer	16.468 (15.07)	5255 (17.77)	< 0.0001	1000 (16.66)	1193 (17.87)		3962 (18.13)		0.0374	
**Antihyperglicemic drugs** (12 months before index AMI hospitalization)										
Insulin	0 (0.00)	10,795 (36.51)	< 0.0001	1052 (17.53)	1263 (18.92)		8480 (50.21)		< 0.0001	
Other AHAs	0 (0.00)	29,392 (99.41)	< 0.0001	5998 (99.95)	6581 (98.56)		16,813 (99.56)		< 0.0001	
**Other medications of interest** (12 months before index AMI hospitalization)										
ACE-I/ARBS	51,901 (47.51)	21,020 (71.10)	< 0.0001	3989 (66.47)	4541 (68.01)		12,490 (73.96)		< 0.0001	
Beta blockers	31,605 (28.93)	14,365 (48.59)	< 0.0001	2774 (46.23)	3057 (45.78)		8534 (50.53)		< 0.0001	
Diuretics	19,686 (17.93)	11,164 (37.75)	< 0.0001	1822 (30.36)	2118 (31.72)		7224 (42.78)		< 0.0001	
Ca-antagonists	24,179 (22.13)	11,459 (38.76)	< 0.0001	1985 (33.08)	2298 (34.42)		7176 (42.49)		< 0.0001	
Lipid lowering drugs	28,353 (25.95)	16,972 (57.40)	< 0.0001	3240 (53.99)	3561 (53.33)		10,171 (60.23)		< 0.0001	
Antiplatelet drugs	33,288 (30.47)	17,071 (57.74)	< 0.0001	3034 (50.56)	3427 (51.33)		10,610 (62.83)		< 0.0001	
Oral anticoagulant drugs	6570 (6.01)	2764 (9.35)	< 0.0001	526 (8.77)	607 (9.09)		1631 (9.66)		0.0890	

ACE-I: angiotensin-converting enzyme inhibitors; AHAs: anti-hyperglycemic agents; AMI: acute myocardial infarction; ARB: angiotensin II receptor agonist blockers; CABG: coronary artery bypass graft; COPD = chronic obstructive pulmonary disease; DM: diabetes mellitus; NSTEMI: non-ST-elevation myocardial infarction; PCI: percutaneous coronary intervention; STEMI: ST-elevation myocardial infarction; SD: standard deviation

**Fig. 2:**
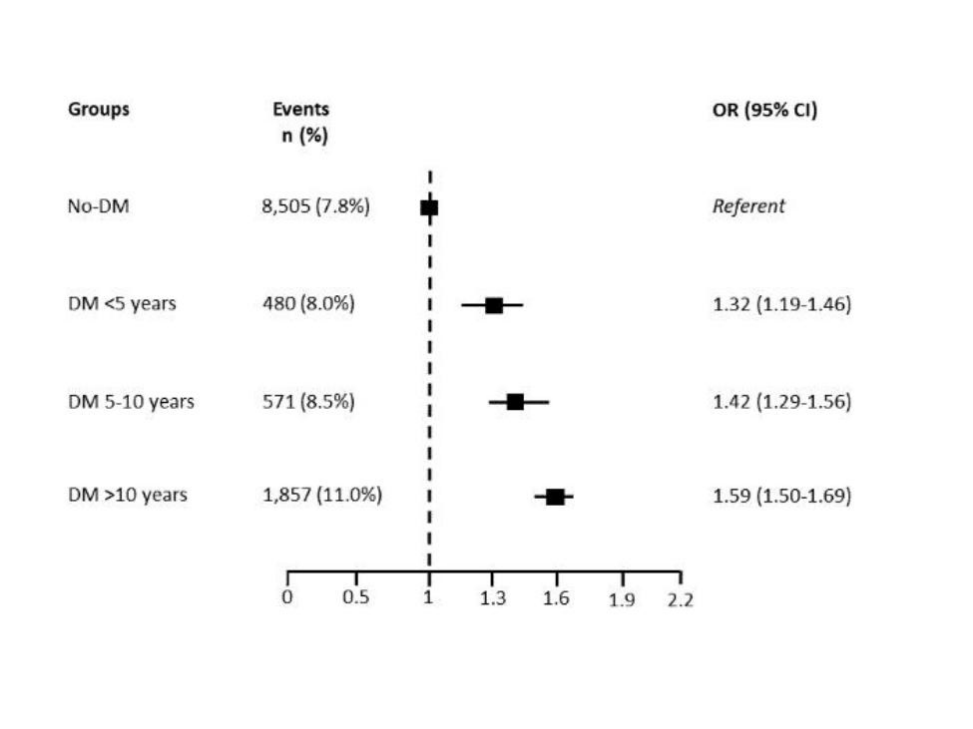
In-hospital mortality rate and adjusted risk in the overall study population according to diabetes mellitus (DM) duration. CI = confidence interval; OR = odds ratio

**Fig. 3:**
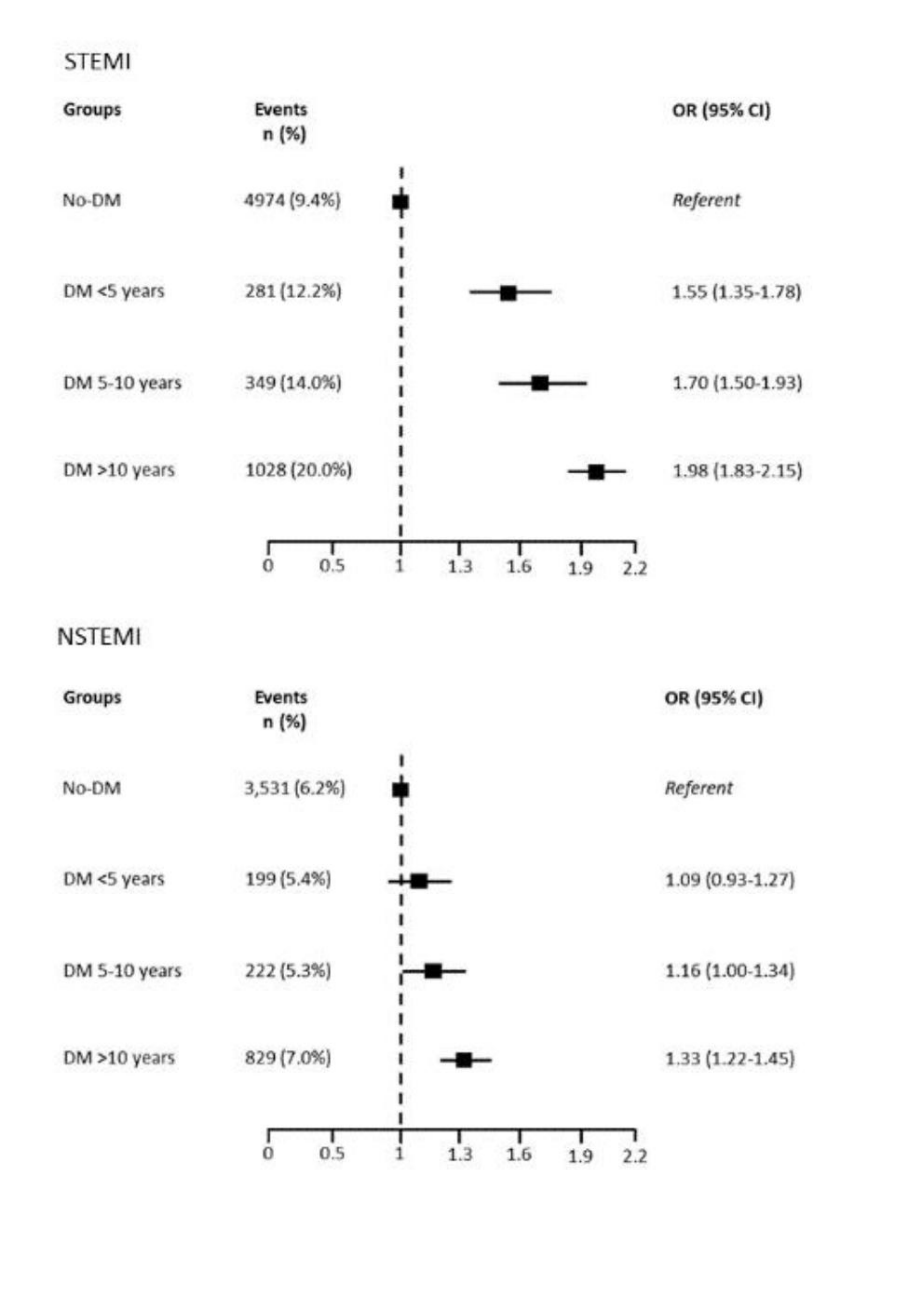
In-hospital mortality rate and adjusted risk in STEMI and NSTEMI patients according to diabetes mellitus (DM) duration. CI = confidence interval; OR = odds ratio

**In-hospital mortality** Overall, in-hospital mortality was 8.2% (n = 11,413); it was 10% (n = 2,908) in patients with DM and 8.0% (n = 8,505) in patients without DM (p < 0.0001) (adjusted OR 1.50, 95% CI 1.43–1.58). Adjusted ORs in STEMI (OR 1.82, 95% CI 1.71–1.95, DM vs. no-DM) and NSTEMI (OR 1.25, 95% CI 1.16–1.34, DM vs. no-DM) patients were considered separately. In Figs. 2 and 3 are reported adjusted ORs according to DM duration.AIC values were 555,959.08 comparing DM presence versus No-DM and 358,330.82 in the model with and without DM and DM duration, indicating that the latter was the best.

**Table 2:** In hospital complications and procedures of patients hospitalized with acute myocardial infarction according to diabetes mellitus status and its duration, from 2010 to 2019

	No-DM Patients No. (109,247)	DM Patients No. (29,566)	P Value	DM Patients (duration of DM) No. (29,566)			P for trend
**Variables**				**< 5 years** No. (6001)	**5–10 years** No. (6677)	**> 10 years** No. (16,888)	
**In-hospital complications**							
Atrial fibrillation	1013 (9.81)	3304 (11.17)	< 0.0001	619 (10.31)	731 (10.95)	1954 (11.57)	0.0238
Cardiogenic shock	3818 (3.49)	1365 (4.61)	< 0.0001	236 (3.93)	250 (3.74)	879 (5.20)	< 0.0001
Acute heart failure	2111 (1.93)	1069 (3.61)	< 0.0001	175 (2.92)	198 (2.97)	696 (4.12)	< 0.0001
Acute renal failure	2163 (1.98)	1086 (3.67)	< 0.0001	143 (2.38)	190 (2.85)	753 (4.46)	< 0.0001
**In-hospital procedures**							
PCI	66,119 (60.52)	16,023(54.19)	< 0.0001	3664 (61.06)	3875 (58.04)	8484 (50.24)	< 0.0001
CABG	2342 (2.14)	643 (2.17)	0.7442	125 (2.08)	168 (2.52)	350 (2.07)	0.1805
Insertion of drug-eluting coronary artery stent(s)	50,594 (46.31)	12,690(42.90)	< 0.0001	2894 (48.23)	3028 (45.35)	6768 (40.08)	< 0.0001
Single coronary vessel PCI	42,841 (39.21)	9760 (33.01)	< 0.0001	2276 (37.93)	2435 (36.47)	5049 (29.90)	< 0.0001
Multivessel PCI	10,470 (9.58)	2974 (10.05)	0.0143	672 (11.20)	666 (9.97)	1636 (9.69)	0.0036
Cardiac retraining	9765 (8.94)	2947 (9.96)	< 0.0001	584 (9.73)	647 (9.69)	1716 (10.16)	0.4383

DM: diabetes mellitus; PCI: percutaneous coronary intervention, CABG: coronary artery bypass graft

In the overall population, the risk of in-hospital mortality increased substantially with the duration of DM in comparison with no-DM patients. Particularly, the highest risk was observed in patients with a history of DM longer than 10 years. This behavior was similar in STEMI and NSTEMI patients.

**1-year mortality.** In the whole study population, cumulative 1-year mortality was 17.8% (n = 24,764) and it was 23% (n = 6,833) in patients with DM and 16% (n = 17,931) in those without DM (p < 0.0001; adjusted HR 1.51, 95% CI 1.46–1.55).

Adjusted HRs in STEMI (HR 1.69, 95% CI 1-62-1.77, DM vs. no-DM) and NSTEMI (HR 1.51, 95% CI 1.45–1.57, DM vs. no-DM) patients were considered separately. In Supplementary Fig. 1 and Fig. 2 are reported adjusted HRs according to DM duration.

The risk of 1-year mortality increased substantially with the duration of DM, with the highest risk observed in patients with DM longer than 10 years. AIC values were 67,252.501 comparing DM presence versus No-DM and 102,740.28 in the model with and without DM and DM duration. indicating that the latter was the best. This occurred both in the overall study population and in STEMI and NSTEMI patients considered separately.

**Fig. 4:**
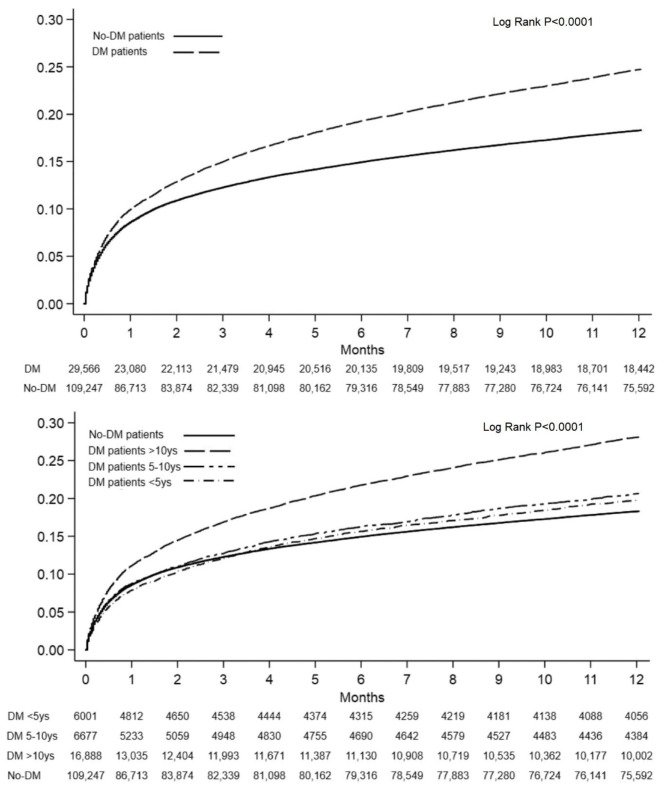
Kaplan-Meier curve analysis of 1-year mortality stratified according to diabetes mellitus status (upper Panel) and its duration (lower Panel) in the overall study population. Log-rank P value < 0.0001

The differences in the cumulative mortality within the first year after AMI between groups are shown in the Kaplan Meyer curves (Fig. 4 and Supplementary Fig. 3). In the overall population, as well as in STEMI and NSTEMI patients, a longer duration of DM was associated with a higher 1-year mortality.

Since no-DM patients had a higher rate of mortality (relative to patients with DM duration < 10 years) in the first three months than in the following period, we performed a sub-analysis on survival in the period starting from three months after hospital admission (where proportionality assumption was met, Supplementary Fig. 4) finding again a rise according to DM duration with respective HRs (95%CIs) for DM < 5, DM 5–10 and DM > 10 relative to no-DM: 1.47 (1.33–1.63), 1.50 (1.36–1.65) and 1.78 (1.67–1.89).

## Discussion

In this analysis from the Lombardy Health Database focusing on a large population of patients hospitalized with AMI, we found that the risk of in-hospital mortality in patients with DM progressively increases in parallel with DM duration.

The mortality of AMI patients has significantly improved over the years with the introduction of PCI and evidence-based medical therapies [1–3]. However, some subgroups of AMI patients still have a less favorable outcome [19]. Among them, patients with DM are at higher risk of mortality than those without DM, irrespective of therapeutic strategies [4]. Accordingly, several clinical risk scores have included the presence of DM among the considered prognostic variables [20, 21]. Nevertheless, it has become increasingly clear that DM encompasses a very heterogeneous population of patients with different risk profiles. This is mainly a function of various factors, including long-term metabolic control, LDL-cholesterol and blood pressure levels, and the number, type, and severity of microvascular and macrovascular complications present in each patient [1, 2, 22]. Although all these complications are closely dependent on the duration of the disease, no study has specifically evaluated the association between DM duration and in-hospital mortality in AMI patients, thus far. Indeed, all the scores currently used for bedside risk stratification in this clinical setting still consider DM only as a binary variable [20, 21]. Thus, we hypothesized that this simple clinical variable − DM duration − by summarizing the burden of DM-related complications, may parallel in-hospital mortality of AMI patients with DM.

To the best of our knowledge, this is the first study evaluating the association between DM duration and in-hospital mortality of patients with AMI. The analysis was conducted on more than 138,000 patients, including almost 30,000 patients with DM, hospitalized with AMI in Lombardy in the last decade and, therefore, treated with current standards of care. We first confirmed a close association between DM presence and increased mortality in AMI patients. This is in line with most previous studies, which consistently reported a similar mortality risk in AMI patients with DM [1–7]. However, this represents the average risk of the entire DM population, which consists of subsets of DM patients with different risk profiles. In particular, we observed that the adjusted risk of in-hospital mortality increases progressively as DM duration increases, with a steep rise in relative risk after 10 years. The detrimental association between DM duration and mortality continues into the year following hospitalization.

However, performing the proportional hazard assumption in the Cox regression analyses we found that the proportionality assumption was met from 3 months to one-year after hospital admission. Although the HRs derived from this analysis were higher than main one (due to the higher rate of mortality in no-DM patients in the first three months) the ordering among the groups remained the same and the differences continued to be highly statistically significant.

The association between DM duration and in-hospital mortality in AMI patients may be explained by the progressive increase in microvascular and macrovascular complication burden that usually parallels disease duration. Indeed, consistently with prior reports, our analysis found that the prevalence of renal disease, peripheral, cerebrovascular and coronary artery disease, and insulin use are associated at baseline with longer DM duration [1–7, 12–15]. Of note, all of them are known to be closely associated with increased in-hospital mortality in patients with AMI, with an additive effect [15–19]. Moreover, a longer DM duration has been previously associated with a greater burden of coronary artery disease, as assessed by coronary angiography [23] and with a higher prevalence of vulnerable coronary plaques [24]. Therefore, it can be inferred that the duration of DM mirrors patients’ frailty during the acute cardiac event.

The finding of a higher mortality risk in STEMI than in NSTEMI patients for the same DM duration does on the one hand reflect the greater hemodynamic impairment typically associated with STEMI, and, on the other hand, support the detrimental interplay among patients’ frailty (DM duration), AMI severity (STEMI vs. NSTEMI), and in-hospital mortality risk in DM patients.

In our study, a disease duration longer than 10 years was associated with the highest risk of death and with an almost two-fold higher incidence of major in-hospital complications, such as acute heart failure, cardiogenic shock, and acute kidney injury. While a DM duration longer than 10 years is a well-known modifier in cardiovascular risk prevention for patients with DM [15], those hospitalized with AMI have traditionally been considered as a single risk category, regardless of disease duration. Thus, our data, focusing on patients hospitalized with AMI, provide unique information by showing heterogeneity in hospital mortality risk among DM patients directly correlated with disease duration. Our data may help physicians refining the early risk stratification of AMI patients with DM and intensify in-hospital therapeutic strategies and post-discharge management of those at higher risk. Therefore, it is advocated that this simple clinical variable be incorporated into the risk scores used in this clinical setting.

### Strengths and limitations

Our study has some strengths and limitations. Administrative databases have been increasingly recognized as a reliable tool to prospectively describe outcomes of large patient’s cohorts representing the real clinical care, since they collect data over time in a standardized way and at low cost [25, 26]. However, limitations that are typical of all the studies based on administrative datasets need to be acknowledged. Administrative data can suffer from systematic biases as their quality depends on the accuracy of coding. However, it should be highlighted that the primary endpoint considered in our study, as well as the variables chosen for risk adjustment, are unlikely to be subject to coding error. Moreover, in our study, DM duration was defined according to chronic exposure to anti-hyperglycemic agents. This does not necessarily reflect the true DM duration because patients might have been treated with lifestyle and diet modifications for years before starting drugs or, despite having DM, they were not taking therapy due to lack of adherence or because they were unaware of the disease (unknown DM). Although we acknowledge the close association between type 1 DM and AMI risk and prognosis [27, 28] we were unable to distinguish between type 1 and type 2 DM, so that both are considered in this study. However, the prevalence of type 1 DM in patients hospitalized with AMI has been reported to be 0.4% when considering all AMI patients and 2% when considering only those with DM [29]. Possibly, in our study this prevalence might have been even lower as patients younger than 50 years were excluded. In addition, some specific pieces of information on clinical variables or laboratory tests, including glycated hemoglobin, body mass index, estimated glomerular filtration rate, and left ventricular ejection fraction, which deserve attention when referring to in-hospital mortality risk in AMI, were not available. Moreover, in our database causes of death are not collected.

## Conclusion

In conclusion, our study demonstrates that the duration of DM parallels in-hospital mortality of DM patients hospitalized with AMI, with the highest mortality risk observed in those with DM duration longer than 10 years. These findings add further evidence to DM duration as an important and simple prognostic risk modifier reflecting the severity of the disease.

## Electronic supplementary material

Below is the link to the electronic supplementary material.

Supplementary Material 1: Table 1S: Baseline characteristics of patients hospitalized with ST-elevation myocardial infarction (STEMI) according to diabetes mellitus status and its duration, from 2010 to 2019. Table 2S: In hospital complications and procedures in patients hospitalized with ST-elevation myocardial infarction (STEMI) according to diabetes mellitus status and its duration, from 2010 to 2019. Table 3S: Baseline characteristics of patients hospitalized with non-ST-elevation myocardial infarction (NSTEMI) according to diabetes mellitus status and its duration, from 2010 to 2019. Table 4S: In hospital complications and in patients hospitalized with non-ST-elevation myocardial infarction (NSTEMI) according to diabetes mellitus status and its duration, from 2010 to 2019.

## Data Availability

The data that support the findings of this study are available from Lombardy Region, but restrictions apply to the availability of these data, which were used under license for the current study, and so are not publicly available. Data are however available from the Lombardy Region upon reasonable request.
