# Supplementary material for: Diabetes mellitus duration and mortality in patients hospitalized with acute myocardial infarction
Source: Cardiovasc Diabetol. 2022 Oct 29;21:223. doi: 10.1186/s12933-022-01655-w (PMC9618227; doi:10.1186/s12933-022-01655-w)
Supplement: Supplementary file 1 — Supplementary Material 1: Table 1S: Baseline characteristics of patients hospitalized with ST-elevation myocardial infarction (STEMI) according to diabetes mellitus status and its duration, from 2010 to 2019. Table 2S: In hospital complications and procedures in patients hospitalized with ST-elevation myocardial infarction (STEMI) according to diabetes mellitus status and its duration, from 2010 to 2019. Table 3S: Baseline characteristics of patients hospitalized with non-ST-elevation myocardial infarction (NSTEMI) according to diabetes mellitus status and its duration, from 2010 to 2019. Table 4S: In hospital complications and in patients hospitalized with non-ST-elevation myocardial infarction (NSTEMI) according to diabetes mellitus status and its duration, from 2010 to 2019. [file 12933_2022_1655_MOESM1_ESM.docx]

**Supplementary Material**

**Diabetes Mellitus Duration and Mortality in Patients Hospitalized with Acute Myocardial Infarction.**

Marta Baviera, Stefano Genovese, Pierluca Colacioppo, Nicola Cosentino, Andreana Foresta, Mauro Tettamanti, Ida Fortino, Maria Carla Roncaglioni, Giancarlo Marenzi

**Abbreviations**

ACE-I: angiotensin-converting enzyme inhibitors

AHAs: anti-hyperglycemic agents

AMI: acute myocardial infarction

ARB: angiotensin II receptor agonist blockers

CABG: coronary artery bypass graft

COPD: chronic obstructive pulmonary disease

DM: diabetes mellitus

NSTEMI: No ST-elevation myocardial infarction

PCI: percutaneous coronary intervention

SD: standard deviation

STEMI: ST-elevation myocardial infarction

**Table 1S.** Baseline characteristics of patients hospitalized with ST-elevation myocardial infarction (STEMI) according to diabetes mellitus status and its duration, from 2010 to 2019.

|  | **No-DM Patients**  No. (52,753) | **DM Patients**  No. (9,937) | **P**  **Value** | **DM Patients**  (duration of DM)  No. (9937) | | | **P**  **for trend** |
| --- | --- | --- | --- | --- | --- | --- | --- |
| **Variables** |  |  |  | **<5 years**  No. (2309) | **5-10 years**  No. (2494) | **>10 years**  No. (5134) |  |
| **Age** (years)**,** mean ± SD | 70.9 ± 11.9 | 70.3 ± 10.2 | <0.0001 | 69.5 ±10.6 | 68.9 ±10.5 | 72.5 ± 9.8 | <0.0001 |
| **Age groups** (years), n (%) |  |  |  |  |  |  |  |
| 50-64 | 18,320 (34.73) | 2878 (28.96) | <0.0001 | 858 (37.16) | 866 (34.72) | 1154 (22.48) | <0.0001 |
| 65-80 | 20,041 (37.99) | 4735 (47.65) |  | 966 (41.84) | 1120 (44.91) | 2649 (51.60) |  |
| >80 | 14,392 (27.28) | 2324 (23.38) |  | 485 (21.00) | 508 (20.37) | 1331 (25.93) |  |
| **Gender** (female) | 17,815 (33.77) | 3843 (38.67) | <0.0001 | 737 (31.70) | 832 (33.36) | 2274 (44.29) | <0.0001 |
| **History of comorbidities,** n (%)  (in the previous 10 years) |  |  |  |  |  |  |  |
| Cerebrovascular disease | 4022 (7.62) | 1525 (15.34) | <0.0001 | 279 (12.08) | 314 (12.59) | 932 (18.15) | <0.0001 |
| Prior myocardial infarction | 1783 (3.38) | 797 (8.02) | <0.0001 | 234 (10.13) | 189 (7.58) | 374 (7.28) | <0.0001 |
| Chronic ischemic heart disease | 4051 (7.68) | 1711 (17.21) | <0.0001 | 321 (13.90) | 350 (14.03) | 1040 (20.26) | <0.0001 |
| Prior PCI or CABG | 3147 (5.97) | 1262 (12.70) | <0.0001 | 305 (13.21) | 300 (12.03) | 657 (12.80) | 0.1451 |
| Chronic heart failure | 544 (1.03) | 280 (2.81) | <0.0001 | 41 (1.78) | 62 (2.49) | 177 (3.45) | <0.0001 |
| Atrial fibrillation | 2480 (4.70) | 799 (8.04) | <0.0001 | 152 (6.58) | 195 (7.82) | 452 (8.80) | 0.0044 |
| Peripheral vascular disease | 1476 (2.80) | 1138 (11.45) | <0.0001 | 131 (5.67) | 217 (8.70) | 790 (15.39) | <0.0001 |
| Lower limb complication | 398 (0.75) | 434 (4.36) | <0.0001 | 51 (2.21) | 62 (2.49) | 321 (6.25) | <0.0001 |
| Renal disease | 1492 (2.83) | 809 (8.14) | <0.0001 | 118 (5.11) | 142 (5.69) | 549 (10.69) | <0.0001 |
| COPD | 2663 (4.29) | 665 (6.69) | <0.0001 | 154 (6.67) | 167 (6.70) | 344 (6.70) | 0.9987 |
| Cancer | 7312 (13.86) | 1700 (17.10) | <0.0001 | 362 (15.68) | 442 (17.72) | 893 (17.39) | 0.2579 |
| **Antihyperglicemic drugs**  (12 months before index AMI hospitalization) |  |  |  |  |  |  |  |
| Insulin | 0 (0.00) | 3101 (31.20) | <0.0001 | 379 (16.41) | 432 (17.32) | 2290 (44.60) | <0.0001 |
| Other AHAs | 0 (0.00) | 9867 (99.29) | <0.0001 | 2308 (99.96) | 2452 (98.32) | 5107 (99.47) | <0.0001 |
| **Other medications of interest**  (12 months before index AMI hospitalization) |  |  |  |  |  |  |  |
| ACE-I/ARBS | 21,701 (41.14) | 6461 (65.01) | <0.0001 | 1420 (61.50) | 1565 (62.75) | 3476 (67.71) | <0.0001 |
| Beta blockers | 11,943 (22.64) | 3850 (38.74) | <0.0001 | 873 (37.81) | 897 (35.97) | 2080 (40.51) | <0.0001 |
| Diuretics | 6927 (13.13) | 2860 (28.78) | <0.0001 | 533 (23.08) | 612 (24.54) | 1715 (33.40) | <0.0001 |
| Ca-antagonists | 9952 (18.87) | 3281 (33.01) | <0.0001 | 648 (28.06) | 752 (30.15) | 1881 (36.64) | <0.0001 |
| Lipid lowering drugs | 10,373 (19.66) | 4684 (47.13) | <0.0001 | 1057 (45.78) | 1104 (44.27) | 2523 (49.14) | <0.0001 |
| Antiplatelet drugs | 11,931 (22.62) | 4560 (45.88) | <0.0001 | 933 (40.41) | 1017 (40.78) | 2610 (50.84) | <0.0001 |
| Oral anticoagulant drugs | 2094 (3.97) | 702 (7.06) | <0.0001 | 135 (5.85) | 174 (6.98) | 393 (7.65) | 0.0186 |

**Abbreviations** ACE-I: angiotensin-converting enzyme inhibitors; AHAs: anti-hyperglycemic agents; AMI: acute myocardial infarction; ARB: angiotensin II receptor agonist blockers; CABG: coronary artery bypass graft; COPD: chronic obstructive pulmonary disease; DM: diabetes mellitus; PCI: percutaneous coronary intervention; SD: standard deviation;.

**Table 2S**. In hospital complications and procedures in patients hospitalized with ST-elevation myocardial infarction (STEMI) according to diabetes mellitus status and its duration, from 2010 to 2019.

|  | **No-DM Patients**  No. (52,753) | **DM Patients**  No. (9937) | **P**  **Value** | **DM Patients**  (duration of DM)  No. (9937) | | | **P**  **for trend** |
| --- | --- | --- | --- | --- | --- | --- | --- |
| **Variables** |  |  |  | **<5 years**  No. (2309) | **5-10 years**  No. (2494) | **>10 years**  No. (5134) |  |
| **In-hospital complications** |  |  |  |  |  |  |  |
| Atrial fibrillation | 4427 (8.39) | 982 (9.82) | <0.0001 | 202 (8.75) | 238 (9.54) | 542 (10.56) | 0.0433 |
| Cardiogenic shock | 2789 (5.29) | 921 (9.26) | <0.0001 | 165 (7.15) | 182 (7.30) | 574 (11.18) | <0.0001 |
| Acute heart failure | 888 (1.68) | 263 (2.64) | <0.0001 | 58 (2.51) | 50 (2.00) | 155 (3.02) | 0.0315 |
| Acute renal failure | 1089 (2.06) | 415 (4.17) | <0.0001 | 57 (2.47) | 79 (3.17) | 279 (5.43) | <0.0001 |
| **In-hospital procedures** |  |  |  |  |  |  |  |
| PCI | 38,329 (72.66) | 6054 (65.45) | <0.0001 | 1708 (73.97) | 1761 (70.61) | 3035 (59.12) | <0.0001 |
| CABG | 1075 (2.04) | 202 (2.03) | 0.9742 | 36 (1.56) | 59 (2.37) | 107 (1.07) | 0.2561 |
| Insertion of drug-eluting coronary artery stent(s) | 28,069 (53.21) | 4884 (49.14) | <0.0001 | 1282 (55.52) | 1287 (51.60) | 2315 (45.09) | <0.0001 |
| Single coronary vessel PCI | 25,818 (48.94) | 4169 (44.65) | <0.0001 | 1099 (47.60) | 1158 (46.43) | 1912 (37.24) | <0.0001 |
| Multivessel PCI | 4816 (9.13) | 925 (9.30) | 0.5697 | 241 (10.44) | 239 (9.58) | 445 (8.67) | 0.0449 |
| Cardiac retraining | 5982 (11.34) | 1089 (10.95) | 0.2713 | 261 (11.30) | 276 (11.07) | 552 (10.75) | 0.7648 |

**Abbreviations**: DM: diabetes mellitus; PCI: percutaneous coronary intervention, CABG: coronary artery bypass graft

**Table 3S.** Baseline characteristics of patients hospitalized with non-ST-elevation myocardial infarction (NSTEMI) according to diabetes mellitus status and its duration, from 2010 to 2019.

|  | **No-DM Patients**  No. (56,494) | **DM Patients**  No. (19,629) | **P**  **Value** | **DM Patients**  (duration of DM)  No. (19,629) | | | **P**  **for trend** |
| --- | --- | --- | --- | --- | --- | --- | --- |
| **Variables** |  |  |  | **<5 years**  No. (3692) | **5-10 years**  No. (4183) | **>10 years**  No. (11.854) |  |
| **Age** (years)**,** mean ± SD | 74.4 ± 11.6 | 71.7 ± 9.6 | <0.0001 | 72.1 ± 10.2 | 70.6 ± 9.7 | 72.5 ± 8.8 | <0.0001 |
| **Age groups** (ys), n (%) |  |  |  |  |  |  |  |
| 50-64 | 13,056 (23.11) | 4546 (23.15) | <0.0001 | 945 (25.60) | 1232 (29.45) | 2369 (20.15) | <0.0001 |
| 65-80 | 21,790 (38.57) | 10,546 (54.73) |  | 1757 (47.59) | 2103 (50.27) | 6686 (56.88) |  |
| >80 | 21,648 (38.32) | 4537 (23.12) |  | 990 (26.81) | 848 (20.27) | 2699 (22.96) |  |
| **Gender** (female) | 22,279 (39.44) | 7407 (37.73) | <0.0001 | 1120 (33.04) | 1479 (35.36) | 4808 (40.91) | <0.0001 |
| **History of comorbidities,** n (%)  (in the previous 10 years) |  |  |  |  |  |  |  |
| Cerebrovascular disease | 6557 (11.61) | 3976 (20.25) | <0.0001 | 589 (15.95) | 714 (17.07) | 2673 (22.74) | <0.0001 |
| Prior myocardial infarction | 3152 (5.58) | 3492 (17.79) | <0.0001 | 735 (19.91) | 682 (16.30) | 2075 (17.65) | <0.0001 |
| Chronic ischemic heart disease | 9269 (16.41) | 6596 (33.60) | <0.0001 | 1049 (28.41) | 1229 (29.38) | 4318 (36.74) | <0.0001 |
| Prior PCI or CABG | 6692 (8.79) | 5109 (26.02) | <0.0001 | 903 (24.46) | 1017 (24.31) | 3189 (27.13) | <0.0001 |
| Chronic heart failure | 1116 (1.98) | 926 (4.71) | <0.0001 | 145 (3.93) | 152 (3.63) | 629 (5.35) | <0.0001 |
| Atrial fibrillation | 4914 (8.70) | 2276 (11.59) | <0.0001 | 394 (10.67) | 489 (11.69) | 1393 (11.85) | <0.0044 |
| Peripheral vascular disease | 2846 (5.04) | 3608 (18.38) | <0.0001 | 410 (11.11) | 545 (13.03) | 2653 (22.57) | <0.0001 |
| Lower limb complication | 577 (1.02) | 1167 (5.94) | <0.0001 | 112 (3.03) | 135 (3.23) | 920 (7.83) | <0.0001 |
| Renal disease | 3089 (5.47) | 2610 (13.54) | <0.0001 | 299 (8.10) | 377 (9.01) | 1934 (16.45) | <0.0001 |
| Chronic obstructive disease | 3946 (6.98) | 1840 (9.37) | <0.0001 | 348 (9.43) | 376 (8.99) | 1116 (9.49) | 0.4201 |
| Cancer | 9156 (16.21) | 3568 (18.17) | <0.0001 | 648 (17.28) | 751 (17.95) | 2169 (18.29) | 0.0374 |
| **Antihyperglicemic drugs**  (12 months before index AMI hospitalization) |  |  |  |  |  |  |  |
| Insulin | 0 (0.00) | 7694 (39.19) | <0.0001 | 673 (18.23) | 831 (19.87) | 6190 (52.66) | <0.0001 |
| Other AHAs | 0 (0.00) | 19.525 (99.47) | <0.0001 | 3690 (99.95) | 4129 (98.71) | 11.706 (99.59) | <0.0001 |
| **Other medications of interest**  (12 months before index AMI hospitalization) |  |  |  |  |  |  |  |
| ACE-I/ARBS | 30,200 (53.46) | 14.649 (74.62) | <0.0001 | 2659 (69.58) | 2976 (71.15) | 9014 (76.69) | <0.0001 |
| Beta blockers | 19,662 (34.80) | 10.515 (53.56) | <0.0001 | 1901 (51.49) | 2160 (51.64) | 6454 (54.91) | <0.0001 |
| Diuretics | 12,659 (22.41) | 8304 (42.30) | <0.0001 | 1289 (34.91) | 1506 (36.00) | 5509 (46.87) | <0.0001 |
| Ca-antagonists | 14,227 (25.18) | 8178 (41.66) | <0.0001 | 1337 (36.21) | 1546 (36.96) | 5295 (45.04) | <0.0001 |
| Lipid lowering drugs | 17,980 (31.83) | 12.288 (62.60) | <0.0001 | 2183 (59.13) | 2457 (58.74) | 7648 (65.07) | <0.0001 |
| Antiplatelet drugs | 21,357 (37.80) | 12.511 (63.73) | <0.0001 | 2101 (56.91) | 2410 (56.71) | 8000 (68.06) | <0.0001 |
| Oral anticoagulant drugs | 4476 (7.92) | 2062 (10.50) | <0.0001 | 391 (10.59) | 433 (10.35) | 1238 (10.53) | 0.0809 |

**Abbreviations** ACE-I: angiotensin-converting enzyme inhibitors; AHAs: anti-hyperglycemic agents; AMI: acute myocardial infarction; ARB: angiotensin II receptor agonist blockers; CABG: coronary artery bypass graft; COPD: chronic obstructive pulmonary disease; DM: diabetes mellitus; PCI: percutaneous coronary intervention; SD: standard deviation.

**Table 4S.** In hospital complications and in patients hospitalized with non-ST-elevation myocardial infarction (NSTEMI) according to diabetes mellitus status and its duration, from 2010 to 2019.

|  | **No-DM Patients**  No. (56,494) | **DM Patients**  No. (19,629) | **P**  **Value** | **DM Patients**  (duration of DM)  No. (19,629) | | | **P**  **for trend** |
| --- | --- | --- | --- | --- | --- | --- | --- |
| **Variables** |  |  |  | **<5 years**  No. (3692) | **5-10 years**  No. (4183) | **>10 years**  No. (11.854) |  |
| **In-hospital complications** |  |  |  |  |  |  |  |
| Atrial fibrillation | 6286 (11.13) | 2322 (11.82) | 0.8203 | 417 (11.29) | 493 (11.79) | 1412 (12.01) | 0.4968 |
| Cardiogenic shock | 1029 (1.82) | 444 (2.26) | <0.0001 | 71 (1.92) | 68 (1.63) | 305 (2.59) | 0.0004 |
| Acute heart failure | 1223 (2.16) | 806 (4.10) | <0.0001 | 117 (3.17) | 148 (3.54) | 541 (4.60) | <0.0001 |
| Acute renal failure | 1074 (1.90) | 671 (3.41) | <0.0001 | 86 (2.33) | 111 (2.65) | 474 (4.03) | <0.0001 |
| **In-hospital procedures** |  |  |  |  |  |  |  |
| PCI | 27,790 (49.19) | 9519 (48.49) | 0.0926 | 1956 (52.98) | 2114 (50.54) | 5449 (46.36) | <0.0001 |
| CABG | 1267 (2.24) | 441 (2.25) | 0.9743 | 89 (2.41) | 109 (2.60) | 243 (2.07) | 0.2005 |
| Insertion of drug-eluting coronary artery stent(s) | 22,525 (39.87) | 7806 (39.76) | 0.7980 | 1612 (43.66) | 1741 (41.62) | 4453 (37.88) | <0.0001 |
| Single coronary vessel PCI | 17,023 (30.13) | 5591 (28.48) | <0.0001 | 1177 (31.88) | 1277 (30.53) | 3137 (26.69) | <0.0001 |
| Multivessel PCI | 5654 (10.01) | 2049 (10.43) | 00849 | 431 (11.67) | 427 (10.21) | 1191 (10.13) | 0.0242 |
| Cardiac retraining | 3783 (6.70) | 1858 (9.46) | <0.0001 | 323 (8.75) | 371 (8.87) | 1164 (9.90) | 0.0374 |

**Abbreviations**: DM: diabetes mellitus; PCI: percutaneous coronary intervention, CABG: coronary artery bypass graft

**Figure 1S.** Cumulative 1-year mortality rate and adjusted risk in the overall study population according to diabetes mellitus (DM) duration.





**Abbreviations:** CI: confidence interval; HR: hazard ratio.

**Figure 2S** Cumulative 1-year mortality rate and adjusted risk in STEMI and NSTEMI patients according to diabetes mellitus (DM) duration.





**Abbreviations:** CI: confidence interval; HR: hazard ratio.

**Figure 3S.** Kaplan-Meier curve analysis of 1-year mortality stratified according to diabetes mellitus status (DM) and its duration in STEMI patients (A and B) and in NSTEMI patients (C and D). Log-rank P values<0.0001.


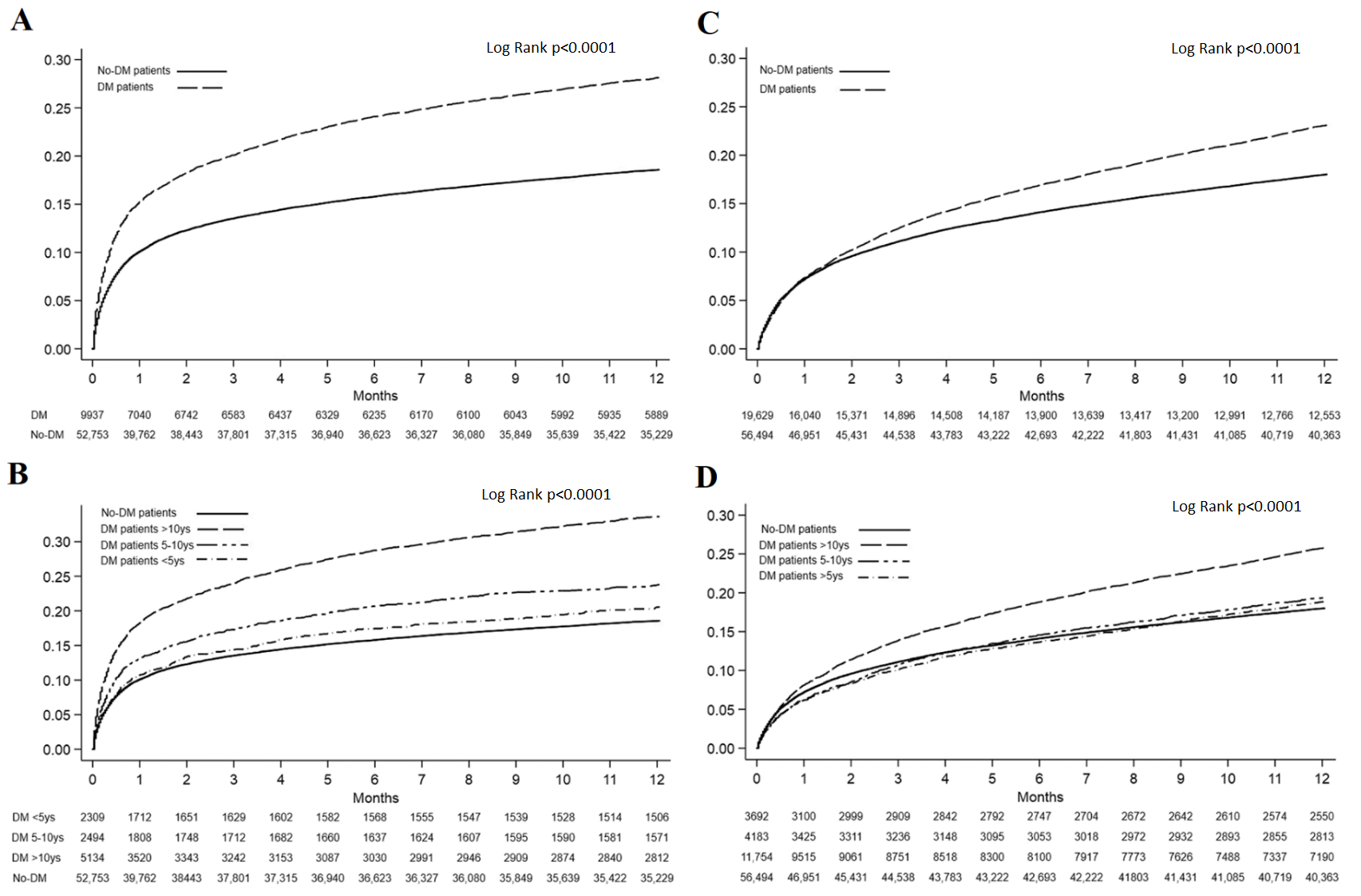


**Figure 4S** Graphical assessment of proportionality assumption in the model with DM duration and no-DM from 3 to 12 months.


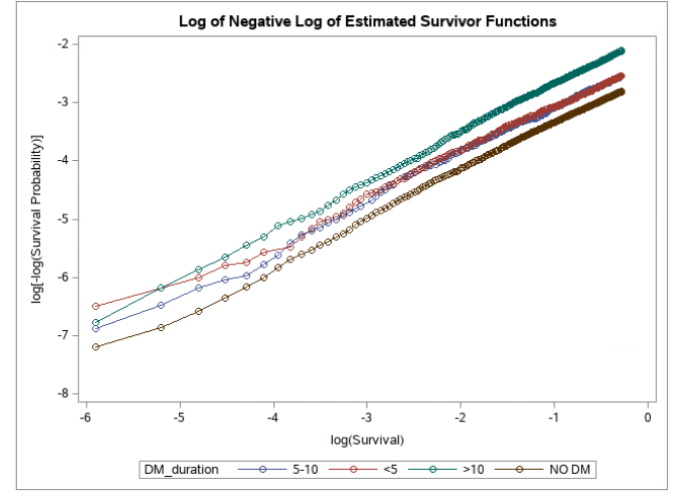


**Appendix**

**International Classification of Disease, Ninth Revision DM (ICD9-CM code) for diagnosis and procedure**

| **Diagnosis and procedures** | **ICD-9-CM Code** |
| --- | --- |
| Myocardial infarction | 410.x |
| Atrial fibrillation | 427.31; 427.32 |
| Cardiogenic shock | 78551 |
| Acute heart failure | 428.21; 428.31; 428.41 |
| Acute renal failure | 584.x |
| Percutaneous coronary intervention | 0066 |
| Coronary arteriography using two catheters | 8856 |
| Insertion of drug-eluting coronary artery stent | 3607 |
| Single coronary vessel or multivessel percutaneous coronary intervention | 0040; 0041 |
| Cardiac retraining | 9336 |
| Cerebrovascular disease | 433.01; 433.11; 433.21; 433.31; 433.81; 433.91; 434.01; 434.91; 430; 431; 432; 432.0; 432.1; 432.9; 435; 435.0; 435.1; 435.2; 435.3; 435.8; 435.9; 436; 433.10; 433.20; 433.30; 433.0; 433.00; 433.1; 433.10; 433.2; 433.20; 433.3; 433.30; 433.8; 433.80; 433.9; 433.90; 434; 434.0; 434.00; 434.9; 434.90; 437; 437.0; 437.1;38.11; 38.12 |
| Chronic ischemic heart disease | 414.x |
| Angina | 411.1; 413; 413.0; 413.1; 413.9 |
| Percutaneous coronary intervention or Coronary artery bypass | 36.x |
| Chronic heart failure | 428.23; 428.33; 428.43;  398.91; 402.01; 402.11; 402.91; 404.01; 404.03; 404.11; 404.13; 404.91; 404.93 |
| Essential hypertension | 4019 |
| Hypertension cardiomyopathy | 402.x |
| Peripheral vascular disease | 440.2; 440.20; 440.21; 440.22; 440.23; 440.24; 440.29;  440.3; 440.30; 440.3; 440.32; 443.81; 250.7; 250.70; 250.71; 250.72; 250.73; 39.50; 38.18; 38.08; 39.90; 39.25; 39.26; 39.29 |
| Lower limb complication | 736 .70; 707.1; 440.23; 707.1; 84.13; 84.15; 84.17; 84.11; 84.12; 730.06; 730.07; 730.16; 730.17; 730.26;  730.27; 730.86; 730.87; 730.96; 730.97; 681.1; 681.10;  681.9; 682.6; 682.7; 711.97; 785.4; 440.24 |
| Renal disease | 585.x; V451; 39.95; 54.98; V561; V562; V563; V563.1;  V563.2; 38.95; 39.27; 39.42; 39.43; 581.81 |
| Chronic obstructive disease | 491.x;492.x;493.x; 494.x;496.x;518.81; 518.83; 518.84 |
| Cancer | Between 140 and 165, Between 170 and 208, Between 210 and 239, 2592 |

[**Anatomical Therapeutic Chemical Classification**](https://en.wikipedia.org/wiki/Anatomical_Therapeutic_Chemical_Classification_System)

| **Drugs** | **ATC-code** |
| --- | --- |
| Antihypertensive drugs | C02*; C03*; C03; C04*; C07*; C08*, C09*; C10BX03; C10BX09 |
| Insulin | A10A* |
| Other anti-hyperglycemic agents | A10BA*; A10BD01; A10BD02; A10BD03; A10BD05; A10BD07; A10BD08; A10BD10; A10BD11; A10BD13; A10BD14; A10BD15; A10BD16; A10BD17; A10BD18; A10BD20  A10BB*; A10BD01; A10BD02; A10BD04;  A10BD06  A10BX02; A10BX03; A10BX08; A10BD14;  A10BG*; A10BD03; A10BD04; A10BD05;  A10BD06; A10BD09; A10BD12  A10BF*; A10BD17  A10BH*; A10BD07; A10BD08; A10BD09;  A10BD10; A10BD11; A10BD12; A10BD13;  A10BD18; A10BD19; A10BD21; A10BH51  A10BX04; A10BX07; A10BX10; A10BX13;  A10BX14; A10BJ*  A10BK*; A10BX09; A10BX11; A10BX12;  A10BD15; A10BD16; A10BD19; A10BD20;  A10BD21 |
| ACE-I/ARBS | C09*; C10BX10 |
| Beta blockers | C07* |
| Calcium channel blockers | C08 |
| Diuretics | C03 |
| Lipid lowering drugs | C10* |
| Antiplatelet drugs | N02BA01; B01AC*; C10BX08; C10BX02; C10BX05; C10BX01 |
| Anticoagulant drugs | B01AA03; B01AA07;  B01AE07; B01AF01; B01AF02; B01AF03 |

**Abbreviation:** ACE-I: angiotensin-converting enzyme inhibitors; ARBs: angiotensin II receptor agonist blockers
